# Review of Silicon Recovery from Diamond Wire Saw Silicon Powder Waste Based on Hydrometallurgical Process

**DOI:** 10.3390/molecules29235645

**Published:** 2024-11-28

**Authors:** Baoshan Xiong, Shifeng Han, Shicong Yang, Keqiang Xie, Kuixian Wei, Wenhui Ma

**Affiliations:** 1 Faculty of Metallurgical and Energy Engineering/National Engineering Research Center of Vacuum Metallurgy, Kunming University of Science and Technology, Kunming 650093, China; 2 State Key Laboratory of Complex Nonferrous Metal Resources Clean Utilization, Kunming University of Science and Technology, Kunming 650093, China; 3 Silicon Industry and Engineering Research Center of Yunnan Province/Silicon Material Industry Research Institution (Innovation Center) of Yunnan Province, Kunming University of Science and Technology, Kunming 650093, China; 4 School of Engineering, Yunnan University, Kunming 650500, China

**Keywords:** diamond wire saw silicon powder waste, hydrometallurgical purification, removal impurity mechanism, recycling purification cycle

## Abstract

The photovoltaic (PV) industry is developing rapidly to support energy transformation and emission reduction. In the whole PV industry chain, diamond wire saw silicon powder (DWSSP) waste is the most promising secondary resource for recycling high-purity silicon. DWSSP mainly contains metal impurities, and the treatment process based on hydrometallurgy can effectively remove metal impurities. The current DWSSP recovery process was divided into three categories: direct acid leaching, pyrometallurgy followed by acid leaching, and acid leaching followed by pyrometallurgy. This paper gives a comprehensive overview of these three purification processes from the aspects of impurity removal and recovery yield. The results suggest that acid leaching followed by pyrometallurgy is currently the most effective process for removing metal impurities from DWSSP. Moreover, this study underscores the potential for enhancing the purity of reclaimed silicon through the application of external field reinforcement, oxygen-regulated acid leaching, and surfactant-facilitated organic acid leaching and points out the development direction for promoting silicon recovery from DWSSP.

## 1. Introduction

In recent years, fossil fuel reserves are decreasing and facing depletion with the rapid development of the global economy, which has led to problems such as the energy crisis, greenhouse effect, and environment pollution [1,2]. The escalating energy crisis and intensifying greenhouse effect are increasingly concerning, prompting global efforts to seek viable solutions [3,4,5]. The adoption of renewable energy sources as a substitute for the majority of fossil fuels is widely regarded as an effective and rapid solution for the prevailing crisis [6,7,8]. In order to achieve this goal, countries around the world are trying to transform to sustainable and environmentally friendly energy [9].

**Figure 1:**
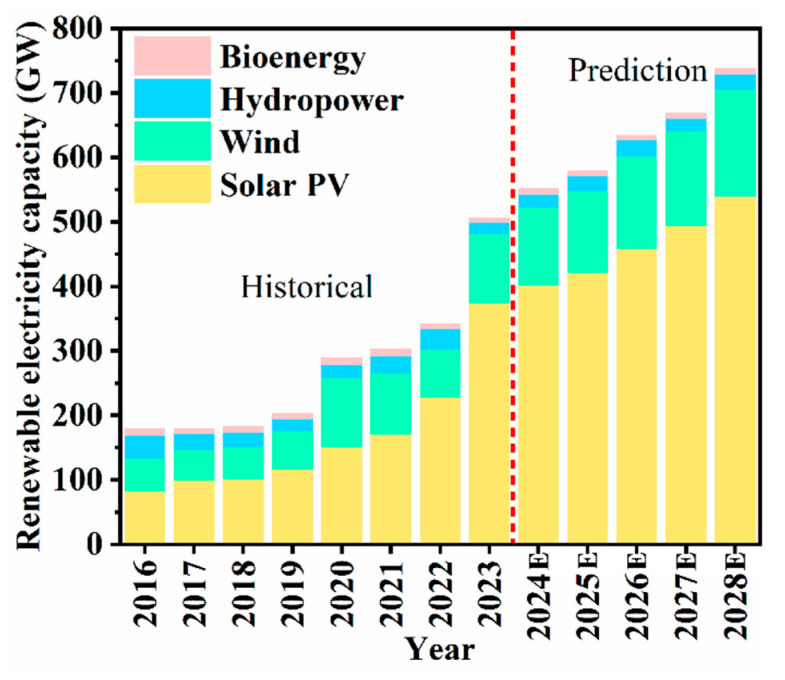
Renewable electricity capacity additions by technology and segment, 2016–2028E.

Utilizing renewable energy sources for electricity generation is essential for mitigating the energy crisis [10]. Solar photovoltaic (PV) is the fastest growing renewable energy industry. As shown in Figure 1, the global solar PV power generation capacity has increased by 146.5 GW in 2023 compared with 2022 [11]. In 2023, renewable energy production in China constituted 50% of the overall power generation output [12]. Solar PV energy is currently experiencing rapid growth within the renewable energy sector and is expected to continue its steady development in the foreseeable future [13].

**Figure 2:**
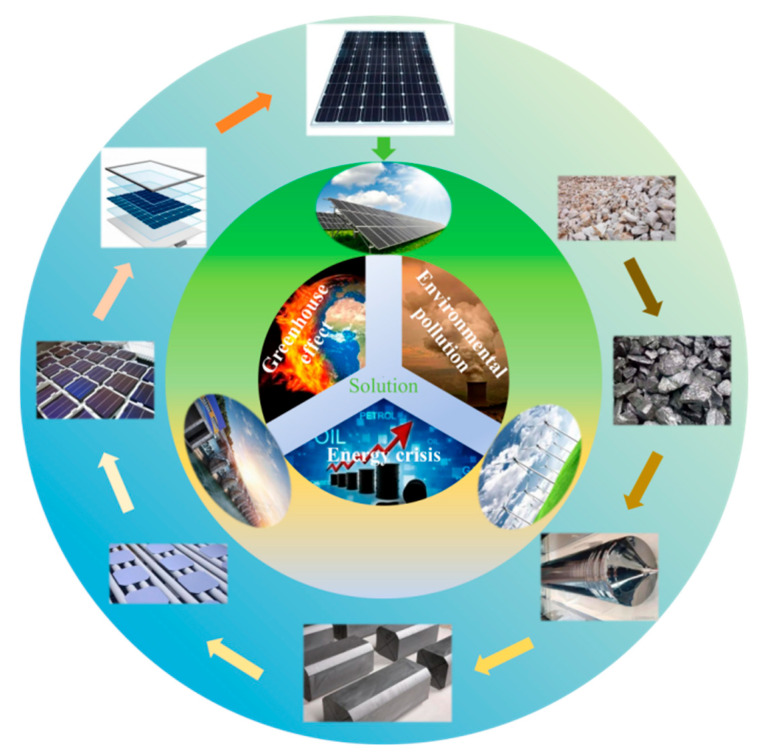
The development of PV industry chain to solve the greenhouse effect, energy crisis, and environmental pollution.

In terms of renewable energy, solar energy is the optimal energy for producing electricity [14]. Solar energy has become an important part of human energy utilization with the decrease in fossil energy [15]. Among all the solar power generation technologies, PV power generation is the most promising route [16,17,18]. Silicon-based solar cells occupy a dominant status in the PV market due to their high efficiency, low manufacturing cost, and outstanding stability [19]. More than 95% of the global PV market depends on crystalline silicon-based solar cells [20,21]. Therefore, as the most widely used material in the PV industry, the global demand for silicon is increasing. As shown in Figure 2, the current silicon market is still highly dominated by monocrystalline silicon wafers [22]. In the PV industry, metallurgical-grade silicon is obtained by smelting silicon stone, after which polycrystalline silicon is obtained via the Siemens process, and monocrystalline silicon rods are obtained by pulling crystals. Monocrystalline silicon wafers are obtained by cutting the truncated monocrystalline silicon rods with a diamond wire cutting machine. Finally, monocrystalline silicon solar PV modules are manufactured through a series of processes. The whole PV industry chain is a highly collaborative and interdependent system. The optimization and upgrading of the purification and recovery process of diamond wire saw silicon powder (DWSSP) in the PV industry can not only promote the rapid development of the PV industry but also provide support for the transformation of the global energy structure. In the year 2023, the consumption of crystalline silicon was about 1.94 million tons all over the world, and the waste of DWSSP is estimated to be about 679,000–873,000 tons. The current price of crystalline silicon is about USD 30,000/t, and it can be calculated that more than USD 20.37 billion of Si was wasted [23]. PV, as the main energy source combined with wind and hydropower generation, can replace the electricity generated by most fossil fuels and plays a vital role in alleviating the energy crisis, greenhouse effect, and environmental pollution [24]. This also confirms the importance of the PV industry and the promotion of sustainable development of the PV industry.

DWSSP is a waste produced in the process of cutting monocrystalline silicon rods [25]. Due to the high content of high-purity silicon and low content of metal impurities in DWSSP, it has potential for recovery and purification. Furthermore, due to the potential danger of exothermic oxidation of DWSSP, it is highly flammable and prone to dust pollution, which is considered to be a serious threat to the workplace. Meanwhile, the importance of DWSSP as a valuable secondary resource is increasing, and its significance within the PV industry is growing increasingly prominent. Therefore, it is imperative for factories to recycle DWSSP in order to effectively manage silicon resources and uphold environmental conservation efforts. However, due to the easy oxidation of DWSSP, the formed silicon oxide layer wraps metal impurities in it, which affects the efficiency of acid leaching purification, leading to generally low purity and yield of the recovered silicon. The silicon purity obtained through the DWSSP process ranges from 3 N to 4 N, and there have been challenges in achieving high-value regeneration for the preparation of solar-grade silicon (6 N). Solving the oxidation problem of DWSSP in the recovery process to improve the purity and yield of recovered silicon is a hot research direction [26].

The particle size of DWSSP is relatively small, and it contains significant levels of the metal impurities elements. Hydrometallurgy facilitates the extraction of a range of valuable elements and demonstrates a high degree of adaptability to different metallurgical materials. Moreover, it also has the advantages of low cost, simple operation, convenient control, and easy realization of a continuous-automatic production process [27,28]. Therefore, as a pretreatment process, hydrometallurgy is the most promising process for efficient, green recovery of high-purity silicon from DWSSP.

This work aims to provide a comprehensive review of silicon recovery from DWSSP waste based on a hydrometallurgical process. It synthesizes the research progress in the recovery of silicon from DWSSP by hydrometallurgical technology and discusses the technologies and challenges. Moreover, this work summarizes the key issues of hydrometallurgical recovery and purification of DWSSP, which looks forward to the future green and sustainable development direction of the PV industry for DWSSP resource treatment.

## 2. Brief of DWSSP Waste

### 2.1. The Generation Process of DWSSP

**Figure 3:**
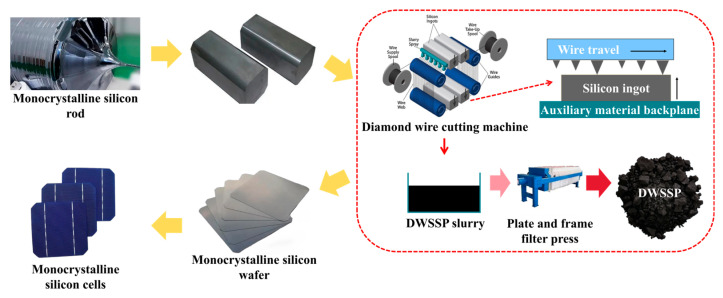
The schematic diagram of DWSSP generation in silicon wafer production [36].

The PV industry chain and the generation process of DWSSP are shown in Figure 3. During the cutting process, approximately 35–45% of the silicon enters the water-based cutting fluid as waste particles [29]. The diamond wire employed for slicing silicon wafers consists of stainless steel coated with a layer of Ni and Fe [30,31]. The organic additives used in the cutting and the auxiliary material backplane of the fixed silicon ingot contain Al [32]. Therefore, during the cutting process, Ni, Fe, and Al impurity elements will enter the DWSSP [33,34]. Due to the use of water-based cutting fluid to cool the diamond wire cutting machine, the cutting fluid will enter the DWSSP to form a slurry, and a flocculant will be added to it in industrial treatment [35]. DWSSP is mainly composed of high-purity silicon particles, water-based cutting fluid, and some metal particles.

### 2.2. Physicochemical Properties of DWSSP

**Figure 4:**
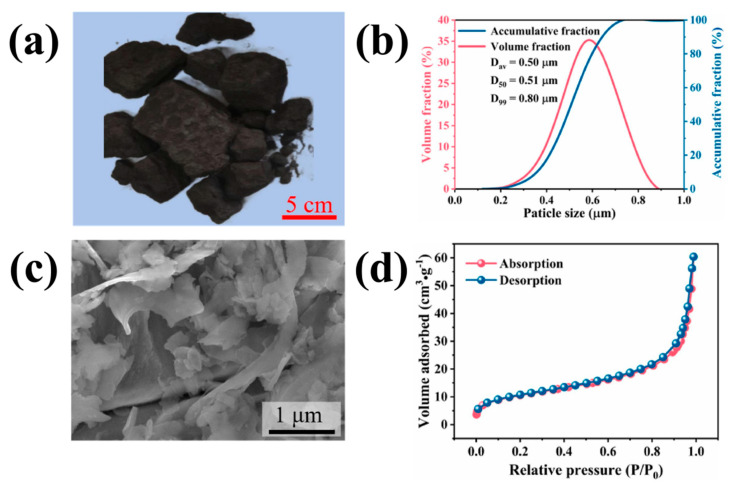
The characterization of raw DWSSP waste: (**a**) the optical image of the cake; (**b**) the particle size distribution; (**c**) the micromorphology; (**d**) the N_2_ adsorption and desorption isotherms [37].

Figure 4 shows the characterization of raw DWSSP waste used by Han et al. Figure 4a is an optical image of the DWSSP waste cake, from which it can be clearly seen that DWSSP is black, granular, and easy to agglomerate. As shown in Figure 4b,c, the DWSSP exhibits a small particle size with a concentrated distribution at the submicron level, featuring an average particle size of 0.5 μm, which is easy to gather together in a slurry. Therefore, particle agglomeration is a problem that cannot be ignored in the leaching process. As shown in Figure 4d, the raw DWSSP waste was found to display type II isothermal adsorption curves, which indicates that there are many pores in DWSSP; the specific surface area is significantly higher than that of the original metal minerals, so the reaction area is also larger than other metal minerals.

The impurity content of five different batches of DWSSP waste are listed in Table 1. Fe, Ni, and Al were determined as the main impurity elements in DWSSP [38]. The contents of all elements in different batches of DWSSP are quite different. The oxygen content in each batch of DWSSP was significantly higher than that of metal impurities. This also shows that the oxidation of DWSSP is an inevitable impurity.

### 2.3. The Micro-Morphology of DWSSP

**Figure 5:**
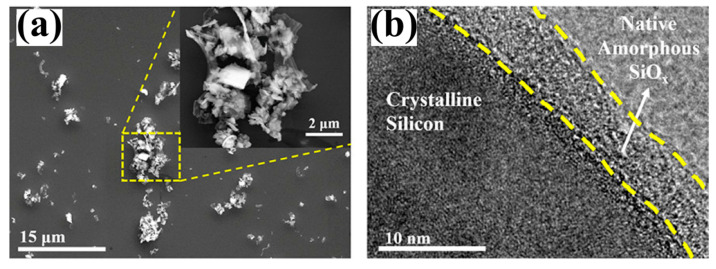
(**a**) SEM image of DWSSP and (**b**) TEM image of DWSSP [45].

As shown in Figure 5, a layer of amorphous SiO*_x_* is coated on the surface of crystalline Si. It indicates that the DWSSP particles are covered by a silicon oxide layer. DWSSP reacts with air or water after its generation though Equations (1) and (2), forming an oxide layer on the bare silicon surface [44].

(1) $$2Si+O_{2}=2SiO_{2}$$

(2) $$Si+2H_{2}O=SiO_{2}+2H_{2}↑$$

The SiO_2_ layer will hinder the process of impurities removal by acid leaching [46]. Moreover, the oxygen element will be transferred during the pyrometallurgical treatment process, resulting in the oxidation of metal impurities [47,48]. The residual oxygen in silicon will reduce the electrical properties of the silicon wafer, thus greatly reducing the power generation efficiency of solar cells [49]. In summary, oxygen affect the silicon product properties in the whole PV industry chain, which is a key issue that must be resolved.

Therefore, the essential factor in silicon recovery from DWSSP waste for the purpose of producing high-purity silicon lies in the removal of metal impurities and the management and reduction of oxygen levels during the acid leaching pretreatment process.

## 3. Hydrometallurgy Purification of DWSSP

The hydrometallurgical process is the crucial pretreatment in the DWSSP recovery and purification process [50,51]. Based on different strategic foci, the process can be classified into three routes: direct acid leaching, pyrometallurgy after acid leaching pretreatment, and pyrometallurgy followed by acid leaching. Although the main goal of these three processes is to remove the impurities from DWSSP, the process routes and the final process products are different. The main purpose of the direct acid leaching process is to first remove metal impurities from DWSSP and then use the purified silicon powder as the raw material for the subsequent process. The aim of acid leaching after pyrometallurgical treatment is to remove impurities through pyrometallurgy, thereby promoting subsequent acid leaching to obtain a high-purity silicon pellet. After acid leaching treatment, most of the metal impurities in DWSSP are removed, and pyrometallurgical treatment is then carried out to remove the residual impurities and solidify the remainder to obtain a silicon casting product.

### 3.1. Direct Acid Leaching Process

**Figure 6:**
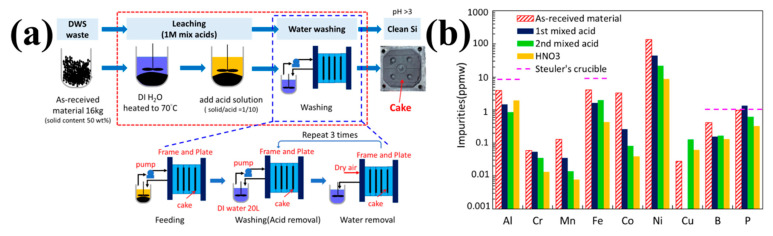
(**a**) The process flow diagram; (**b**) impurity content after acid leaching [39].

Direct acid leaching is a process that can effectively remove metal impurities [52]. Yang et al. used the HCl + H_2_SO_4_ + HF acid leaching system to leach DWSSP and then treated it with HNO_3_. The purity of the recovered silicon was nearly 5 N [39]. The process flow diagram is shown in Figure 6a. As shown in Figure 6b, since HNO_3_ treatment will increase the oxygen content of DWSSP, the best process should be to further remove Ni via nitric acid treatment, and then eliminate the oxide layer with HF treatment. With the HCl + H_2_SO_4_ + HF acid leaching system, more than 94% of the metals could be removed. The addition of HNO_3_ can further improve the removal efficiency of Ni and promote hydrophilicity in particles for improved handling. However, this process results in a significant increase in oxygen content, reaching nearly 10 wt%, which leads to more Si elements being oxidized to SiO_2_, increasing the loss of Si. Although this acid leaching system is effective for dissolving metal impurities, the presence of HF exacerbates silicon loss and does not fully address the oxidation issues. The oxygen content in the final silicon cake far exceeds the requirements of solar-grade silicon.

In order to intuitively compare the removal effects of different acid leaching systems on metal impurities, the process conditions of various direct acid leaching processes and the removal efficiency of metal impurities are shown in Table 2. The optimum range of parameters such as temperature, liquid–solid ratio, and time of acid leaching have been basically determined. At present, the type of acid leaching system has the greatest influence on the removal efficiency of metal impurities. The HCl + HF system currently shows the best metal impurity removal effect.

**Figure 7:**
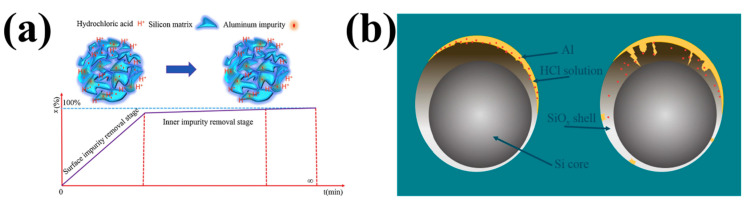
Schematic diagrams of Al dissolution behavior: (**a**) the kinetic mechanism diagram of the homogeneous model [53]; (**b**) the kinetic mechanism diagram of the Avrami model [54].

Yang et al. studied the kinetic mechanism of Al removal by HCl [53]. They found that the dissolution mechanism of Al conforms to the Avrami model during HCl leaching. As shown in Figure 7, the dissolution behavior of metal impurities strongly depends on the spatial distribution characteristics of the silicon matrix. The process of Al dissolution occurs in two distinct stages. Initially, the majority of the Al located on the external surface of DWSSP undergoes dissolution, then the internal Al impurities are mainly dissolved by hydrogen ions through the channel formed by the dissolution of the outer surface Al into the interior. Conversely, if Al does not make contact with the external surface, the dissolution process is governed by diffusion and ceases, preventing the formation of any channel. Yang et al. concluded that the homogeneous reaction model is more suitable for describing the first stage of Al dissolution, and the Avrami model is more suitable for describing the second stage of Al dissolution [54]. This indicates that the removal efficiency is higher closer to the surface of DWSSP and the removal efficiency is lower further from the surface of DWSSP.

**Table 2:** The process conditions of various direct acid leaching processes and the removal efficiency of metal impurities.

Author	Leaching System	S/L	Temperature	Time	Removal Efficiency	Ref.
Yang et al.	1 M (HCl + H_2_SO_4_ + HF)	1/10	343 K	9 h	≥94%	[39]
Yang et al.	4 M HCl	1/10	343 K	3 h	95.6%(Al)	[53]
Zhang et al.	3.6 M HCl	1/15	333 K	1.6 h	98.37%(Fe)	[55]
Kong et al.	H_2_SO_4_	1/10	333 K	1 h	94.34%(Fe)	[56]
Yang et al.	4 M HCl +0.5 M HF	1/10	333 K	3 h	99.28%	[57]
Yang et al.	4 M HCl and 2 M HCl + 2.5 M HF	1/10	333 K	3 h	99.85%	[58]

**Figure 8:**
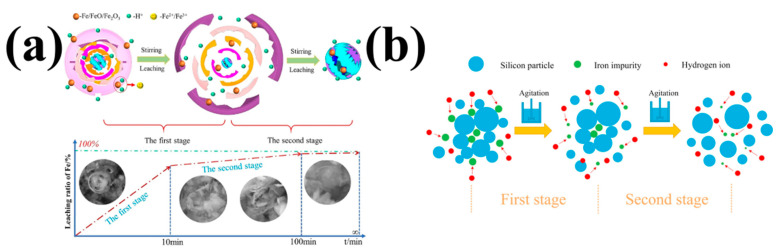
(**a**) The kinetic mechanism diagram of the homogeneous model [55]; (**b**) the kinetic mechanism diagram of the heterogeneous model [56].

As shown in Figure 8a, Zhang et al. studied the kinetic mechanism of Fe removal by HCl [55]; the removal of Fe in DWSSP is also divided into two stages. However, the authors believe that DWSSP will agglomerate together to form a hard caking structure so that the structure cannot be broken by stirring, and the leaching of Fe is affected. The Fe element is mainly distributed on the surface of DWSSP, which should be easily removed in theory. The Fe encapsulated in DWSSP aggregates can only be removed by destroying the DWSSP aggregates.

As shown in Figure 8b, Kong et al. investigated the removal of Fe from DWSSP through H_2_SO_4_ leaching. The kinetic mechanism of Fe removal was also investigated with heterogeneous and homogeneous reaction methods. The removal of Fe in DWSSP can be divided into two stages. The two stages are the same as those described in Figure 8a. Both stages conform to the homogeneous kinetic model. The efficiency of removing Fe in DWSSP by H_2_SO_4_ treatment is significant; it can be seen that the Fe is mainly concentrated on the surface of DWSSP [56]. It was proven that the distribution of Fe and Al is similar. These two metal impurities are easily removed.

**Figure 9:**
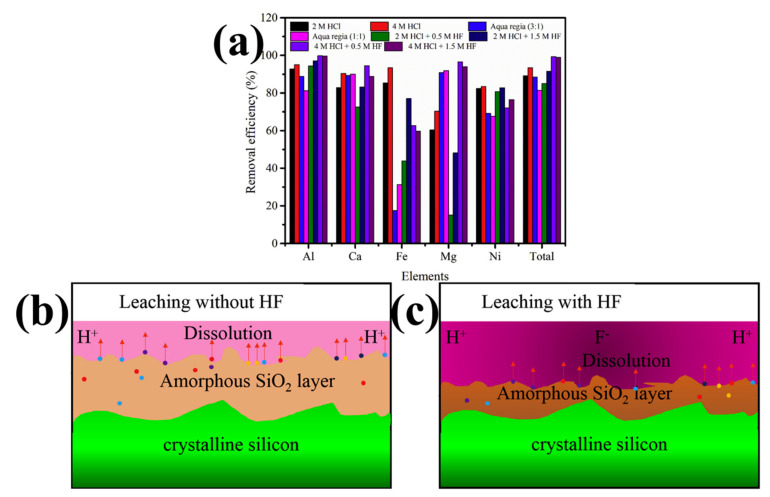
(**a**) The removal efficiency of impurities in an acid leaching experiment; the mechanism schematic diagram of impurity removal. (**b**) Single HCl leaching; (**c**) HCl + HF mixture leaching [57].

As shown in Figure 9, Yang et al. used HF + HCl to leach DWSSP and found that HF has a better effect on impurities under the conditions of 4 M HCl. Al and Fe are mainly distributed on the surface of DWSSP [57]. The removal efficiency of Ca and Mg in the mixed acid leaching system of 4 M HCl and 0.5 M HF was significantly higher than that in the single HCl leaching, and this is because HF can corrode the SiO_2_ layer. The study showed that the Al and Ca contaminants entangled in DWSSP exist in an ionic state. Although the removal efficiency of HF is high, the yield of recovered silicon will decrease.

(3) $$4HF+SiO_{2}=SiF_{4}↑+2H_{2}O$$

In order to explore the dissolution behavior of various metal impurities, Yang et al. use a two-stage acid leaching combined process of 4 M HCl and 2 M HCl + 2.5 M HF to treat DWSSP. They found that there are two states of metal impurities in DWSSP. HF can promote the decomposition of the silicon oxide layer, which can also promote the removal of metal impurities. The removal efficiency of total metal impurities reached 94.38% [58]. The leaching effect of the mixed acid leaching system is obviously better than that of single acid leaching. HF is an indispensable inorganic acid in the mixed acid system because it can react with silicon oxide and has the potential to significantly improve the removal efficiency of metal impurities. However, the introduction of HF may lead to hydrophobicity and processing challenges of silicon particles.

### 3.2. Pyrometallurgy Followed by Acid Leaching Process

**Figure 10:**
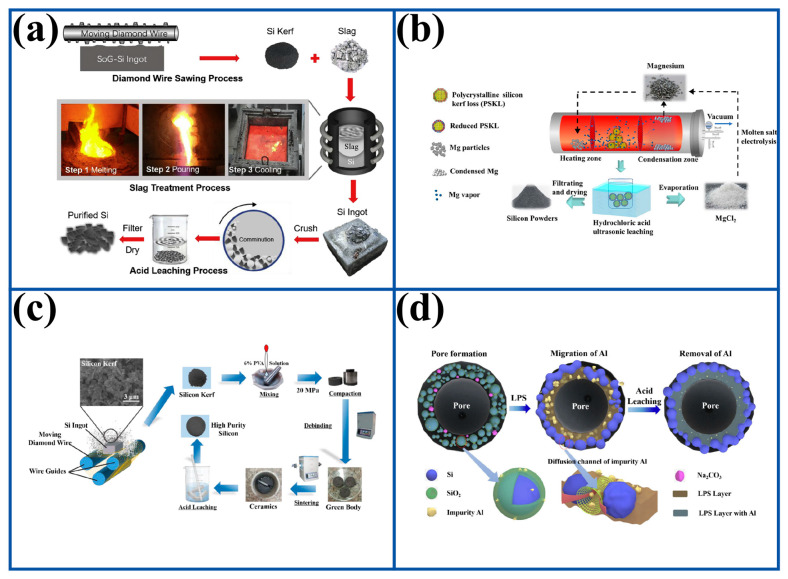
(**a**) Na_2_O-SiO_2_ slag treatment–acid leaching process [40]; (**b**) vacuum magnesium thermal reduction–hydrochloric acid leaching process [41]; (**c**) two-step sintering–acid leaching process [59]; (**d**) Na_2_CO_3_-assisted pressure-less sintering–acid leaching process [60].

As shown in Figure 10a, Huang et al. first treated DWSSP with Na_2_O-SiO_2_ slag and then leached with 2 M HCl. The refining temperature was 2023 K, and the refining time was 3 h [40]. At 2023 K, the affinity of Ni to O is lower than that of Na, and the affinity of other impurity elements to O is higher than that of Na. Therefore, the content of Fe, Al, Ca, B and P would decrease after slag treatment, but the content of Fe is almost unchanged. The efficiency of removing Ca, Ni, and B from DWSSP by acid leaching is poor, but the removal efficiency of Ca, Ni, and B saw significant improvements after slag treatment and acid leaching. The total removal efficiency of Fe, Al, Ca, Ni, B, and P reached 99.2% and the purity of recovered silicon increased from 93.3% to 99.98% after 12 h of leaching with 2 M HCl after slag treatment. It can be speculated that most of the elements are wrapped in DWSSP by SiO_2_ layer. The Na_2_O-SiO_2_ slag can effectively protect DWSSP from oxidation during melting. However, the DWSSP after slag treatment is in the form of a silicon ingot, the recovery rate of silicon only reaches 63.2%, and it needs to be crushed before subsequent acid leaching. This is not conducive to the removal of metal impurities but also makes the removal process longer, increasing the oxidation degree of DWSSP.

As shown in Figure 10b, Yang et al. used vacuum magnesium thermal reduction to treat DWSSP and then leached it with 4 M HCl [41]. In the vacuum magnesium thermal reduction process, the Mg vapor is used as the reductant to reduce the amorphous SiO_2_ in DWSSP and generate MgO. The reaction is shown in Equation (4). Thereafter, the produced MgO is leached by using the HCl to eliminate oxygen and recover Si.

(4) $$2Mg+SiO_{2}=2MgO+Si$$

After reduction treatment at 923 K for 30 min, DWSSP was treated by 4 M HCl at 343 K for 6.5 h at a liquid–solid ratio of 10:1, and the oxygen removal efficiency and silicon recovery fraction attained 98.43% and 94.46%, respectively. However, the reaction between Mg vapor and SiO_2_ is a gas–solid reaction, and as the kinetic process is controlled by diffusion, SiO_2_ cannot be completely reduced. In the worst case, it will lead to the appearance of Si-SiO_2_-Si structures in DWSSP, which is not conducive to subsequent acid leaching treatment and reduces the yield of recovered silicon. Moreover, Mg_2_Si would react with HCl to produce SiH_4_ gas in the subsequent acid leaching treatment, resulting in a further decrease in the yield of recovered silicon. The maximum removal efficiency of Al and Fe impurities reached 86.72% and 80.4%, respectively. The focus of the research is on deoxygenation, so the removal efficiency of metal impurities is not significant.

As shown in Figure 10c, Zhang et al. used a two-step sintering process to treat DWSSP and then leached with HCl [59]. The introduction of two-step sintering can decompose the silicon oxide layer of the DWSSP and promote densification, thereby inhibiting grain growth. After two-step sintering treatment at 1673 K in the first stage and 1623 K for 10 h in the second stage, DWSSP was treated with 4 M HCl at 298 K for 4 h at a liquid–solid ratio of 10:1, and the silicon yield and the purity of silicon reached 96%. Although the 52.4% oxygen in DWSSP can be removed during the sintering process, there is no oxygen control treatment in the subsequent acid leaching process, and the problem of oxidation still exists. After sintering treatment and acid leaching, the recovery rate of silicon increased from 63.12% to 96.03% and the purity increased from 93.77% to 96.05%; however, the level of purity in the reclaimed silicon falls significantly below the requirement for solar-grade silicon.

As shown in Figure 10d, Zou et al. used Na_2_CO_3_-assisted pressure-less sintering and acid leaching to treat DWSSP [60]. The introduction of Na_2_CO_3_ sintering aids the porosity of DWSSP after low-pressure sintering treatment of DWSSP, which is helpful to increase the contact surface area and improve DWSSP treatment in the subsequent acid leaching process. DWSSP was sintered and then treated with 4 M HCl + 2 M HF, and the impurity removal efficiency of Al and silicon yield reached 99.9% and 76.6%, respectively. The content of Al was as low as 0.07 ppmw. It can be concluded that the Na_2_CO_3_-assisted sintered DWSSP was able to obtain a high removal efficiency of Al and a high yield of silicon product. This is because Na_2_CO_3_ and the SiO_2_ shell of DWSSP form a liquid phase sintering layer during the sintering process. The liquid phase formed during its formation provides a diffusion channel for the Al impurities, and the Al impurities tend to migrate from the SiO_2_ shell to the liquid phase region so that the Al impurities are enriched in the liquid phase sintering layer, which provides favorable conditions for acid leaching.

Although pyrometallurgical treatment can deoxidize and remove impurities, the silicon ingot obtained after pyrometallurgical treatment cannot be used as the final product for 6 N grade silicon preparation, which needs to be broken before subsequent acid leaching treatment. Moreover, the purity of the recovered silicon obtained by pyrometallurgy followed by acid leaching process is still 3–4 N. Compared with the direct acid leaching process, the purity of recovered silicon is reduced by 1–2 N.

### 3.3. Acid Leaching Followed by Pyrometallurgy

The problem of oxygen in the process of acid leaching after pyrometallurgy still exists as oxygen will still be introduced in the subsequent acid leaching process [42]. Therefore, DWSSP was first treated by acid leaching and pyrometallurgy was used as the final treatment process. Acid leaching followed by pyrometallurgy can not only maximize the purity of recovered silicon but also directly obtain silicon ingots [43].

**Figure 11:**
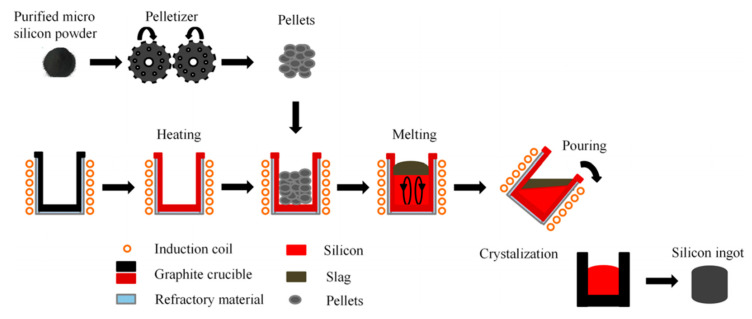
Schematic of DWSSP melting process [61].

Kong et al. first treated DWSSP with H_2_SO_4_. After 25% H_2_SO_4_ leaching at 333 K for 80 min with a liquid–solid ratio of 10:1, the contents of Al, Na, Ca, Fe, and P were reduced to 278 ppmw, 57 ppmw, 237 ppmw, 153 ppmw, and 1.32 ppmw, respectively. Then, DWSSP was induction melted in an air atmosphere at 1773 K, and the purity and yield of the recovered silicon were 99.99% and 58%, respectively [61]. The process flow diagram is shown in Figure 11. This process further improves the purity of the recovered silicon, and a silicon ingot product is obtained. Although the purity of the recovered silicon is close to that of solar-grade silicon, it has not yet reached the standard and still needs to be optimized.

**Figure 12:**
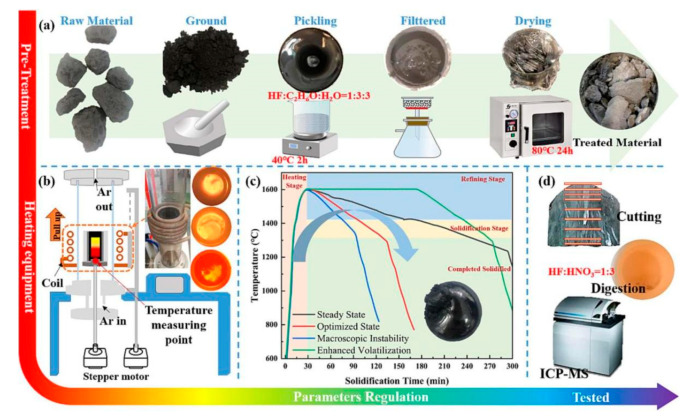
(**a**) Acid pickling pretreatment of DWSSP; (**b**) schematic diagram of induction melting furnace; (**c**) temperature variation curve; (**d**) macro shots of ingots obtained [62].

Hu et al. first treated DWSSP with HF at 40 °C for 2 h and then used heating coupled with directional solidification to refine and purify DWSSP. The analysis revealed that approximately 82.5% of the purified ingots attained a purity level of 5.5 N, and the yield of recovered silicon was 86.89% [62]. The process flow diagram is shown in Figure 12. The thermal control directional solidification technology proposed by Hu et al. can optimize the melt temperature change curve, shorten the temperature fluctuation stage after melt nucleation, stabilize the intergranular structure, and optimize the impurity migration. Furthermore, approximately 82.5% of the purified ingots attained a purity level of 5.5 N. Although the purity does not reach 6 N, it proves the potential of acid leaching followed by pyrometallurgy. After the parameters of acid leaching and pyrometallurgy are optimized, it is expected to achieve the regeneration of 6 N grade silicon.

**Figure 13:**
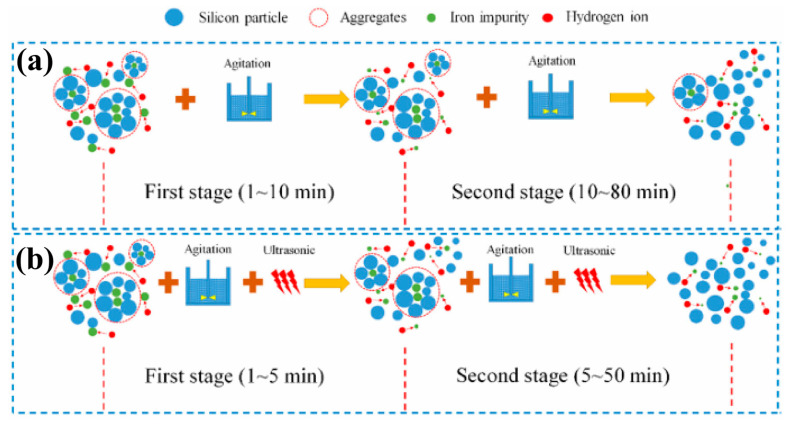
Comparison schematic diagram of iron leaching process during (**a**) conventional and (**b**) ultrasound-assisted leaching [64].

Outside of traditional acid leaching process, researchers are exploring the application of external field intensifying methods to enhance acid leaching and achieve a higher impurity removal efficiency. Two prominent external field strengthening methods being investigated are ultrasonic and microwave heating. Ultrasonic-assisted leaching can effectively shorten the leaching time and improve the leaching efficiency [63]. Kong et al. used ultrasonic-assisted 12% H_2_SO_4_ to leach Fe from DWSSP. The optimum parameters for Fe removal were an ultrasonic frequency of 80 kHz, ultrasonic power of 270 W, reaction temperature of 333 K, and acid concentration of 12%, for which the removal fraction of Fe reached 95.24% [64]. As shown in Figure 13, the results show that ultrasonic is more beneficial in promoting the breakage of DWSSP aggregates than conventional stirring, thereby increasing the leaching efficiency of metals.

Unlike ultrasound, microwave has the characteristics of rapid heating [65]. Hou et al. used microwave-assisted HCl acid leaching to remove impurities in DWSSP [66]. Using microwave-assisted acid leaching, DWSSP can be purified from 98.70% to 99.57% in 10 min under suitable experimental conditions, including hydrochloric acid concentration of 2 M, liquid–solid ratio of 6:1, leaching time of 10 min, leaching temperature of 373 K, and particle size of 98 nm. The reason why the microwave heating effect is good is that compared with traditional heating, microwave-assisted heating is selective. The different microwave absorption capabilities between metal impurities and silicon oxides can cause a temperature difference, which can increase the boiling point of the leaching system and help to improve the leaching efficiency.

Obviously, a silicon ingot product with purity of 4–5 N can be obtained by acid leaching followed by pyrometallurgy. On the basis of this process, the application of microwave heating-assisted acid leaching before pyrometallurgical treatment is expected to further improve the removal efficiency of metal impurities in DWSSP.

## 4. Conclusions and Recommendations

Efficient recovery of solar-grade silicon from DWSSP waste is necessary for promoting the green and sustainable development of the PV industry. Acid leaching followed by pyrometallurgy is the most promising process among the three recovery routes. However, the problems of oxygen and residual metal impurities after acid leaching pretreatment have not been completely solved. Therefore, there is an urgent need to reduce the oxygen and metal in the acid leaching process and establish green and efficient oxygen control recovery from DWSSP for high-purity silicon preparation in this field.

The prospects for solar-grade silicon with 6 N grade recovery and preparation from DWSSP are laid out as follows:

1. At present, the oxygen content of DWSSP is generally high, which greatly affects the efficiency of subsequent hydrometallurgy recovery. Therefore, DWSSP should be managed to control the oxygen from the source generation before recycling.
2. Excessive oxidation of DWSSP should be avoided. Especially in the process of acid leaching, the acid leaching conditions should be properly adjusted to inhibit the oxidation of DWSSP as well as metal removal during the current hydrometallurgical purification pretreatment process.
3. In the process of acid leaching treatment of DWSSP, different acids and external field strengthening should be combined to improve the acid leaching efficiency and reduce the oxidation in order to obtain solar-grade silicon accessibly with a feasible route.

In summary, acid leaching followed by pyrometallurgy is the most efficient process for recovering and purifying DWSSP at present. In order to further improve the purity and yield of recovered silicon, more efficient measures must be taken in acid leaching systems, external field strengthening, and oxygen control methods.

## Figures and Tables

**Table 1:** Impurities content in DWSSP (ppmw).

DWSSP	Compositions								
	Fe	Ni	Al	Ca	Mg	Ti	Mn	O	Ref.
1	40.9	103	3.8	4.9	-	-	0.2	50,000	[39]
2	935	122	5757	150	-	-	-	60,000	[40]
3	1214	57	239	-	84	-	-	160,000	[41]
4	100	-	8700	600	100	700	-	38,500	[42]
5	1100	100	5000	300	-	200	-	94,500	[43]

## Data Availability

No new data were created or analyzed in this study.
